# Supplementary material for: Genome-Wide Characterization and Identification of Auxin Response Factor (ARF) Gene Family Reveals the Regulation of RrARF5 in AsA Metabolism in Rosa roxburghii Tratt. Fruits
Source: Biology (Basel). 2025 Sep 1;14(9):1156. doi: 10.3390/biology14091156 (PMC12467559; doi:10.3390/biology14091156)
Supplement: Supplementary file 1 [file biology-14-01156-s001.zip › Supplemental Figures.pdf]

Title: Genome-wide characterization and identification of ARF gene family reveals the regulation of RrARF5 in AsA metabolism in *Rosa roxburghii Tratt* fruits

Supplementary Data

Figure S1. A comparison of the partial amino acid sequence of RrARFs.

Figure S2. Phylogenetic tree of the MDHAR gene in *Arabidopsis thaliana*.

Figure S3. The cis-acting regulatory elements of *RrMDHAR1* gene in *R. roxburghii*.

Figure S4. Transcription levels of *RrARF5* and *RrMDHAR1* at different fruit ripening stages by RNA-seq analysis.

Figure S5. Hierarchical clustering tree with 14 co-expressed gene modules.

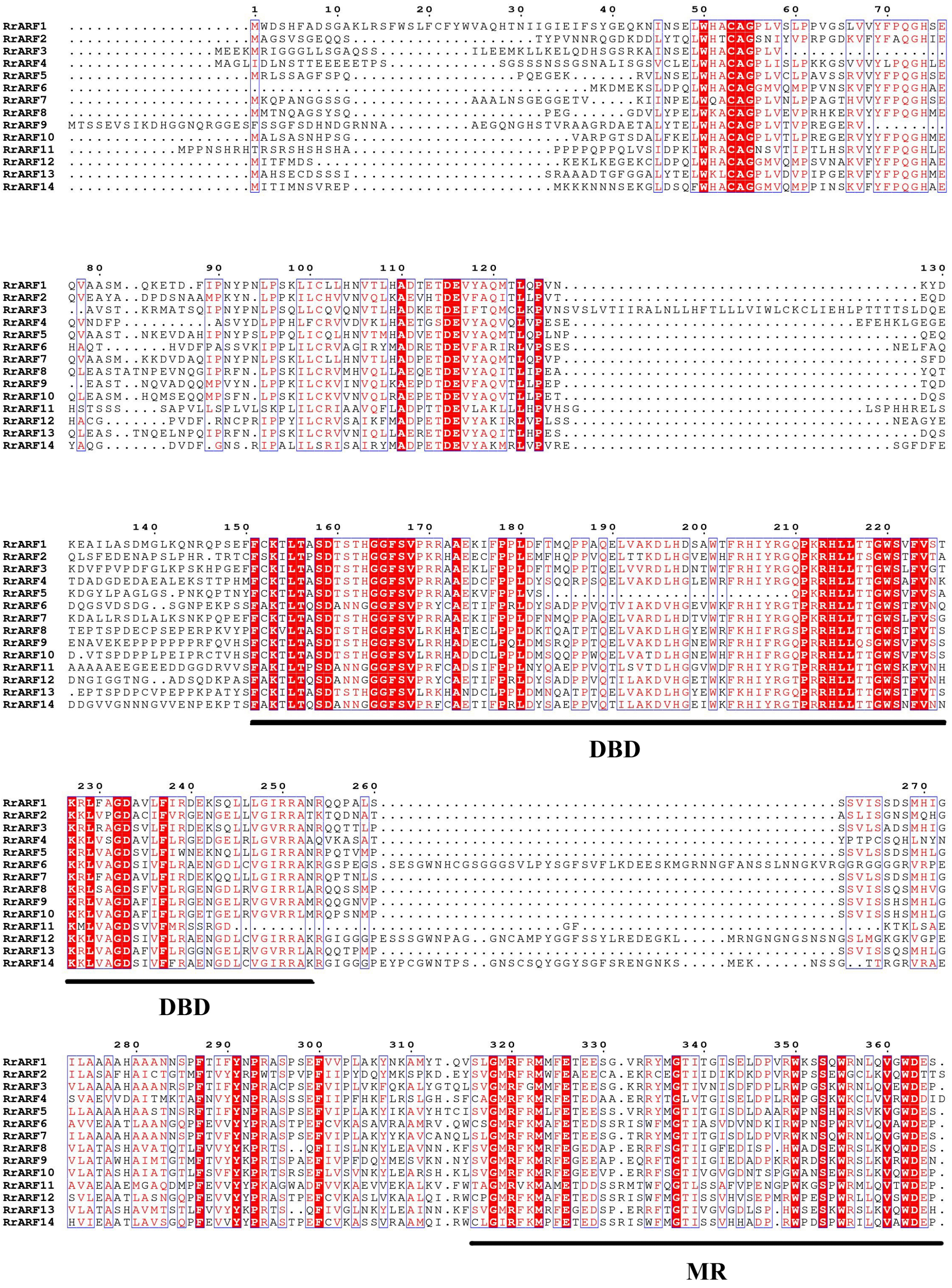
RrARF1
RrARF2
RrARF3
RrARF4
RrARF5
RrARF6
RrARF7
RrARF8
RrARF9
RrARF10
RrARF11
RrARF12
RrARF13
RrARF14
DBD
DBD
MR

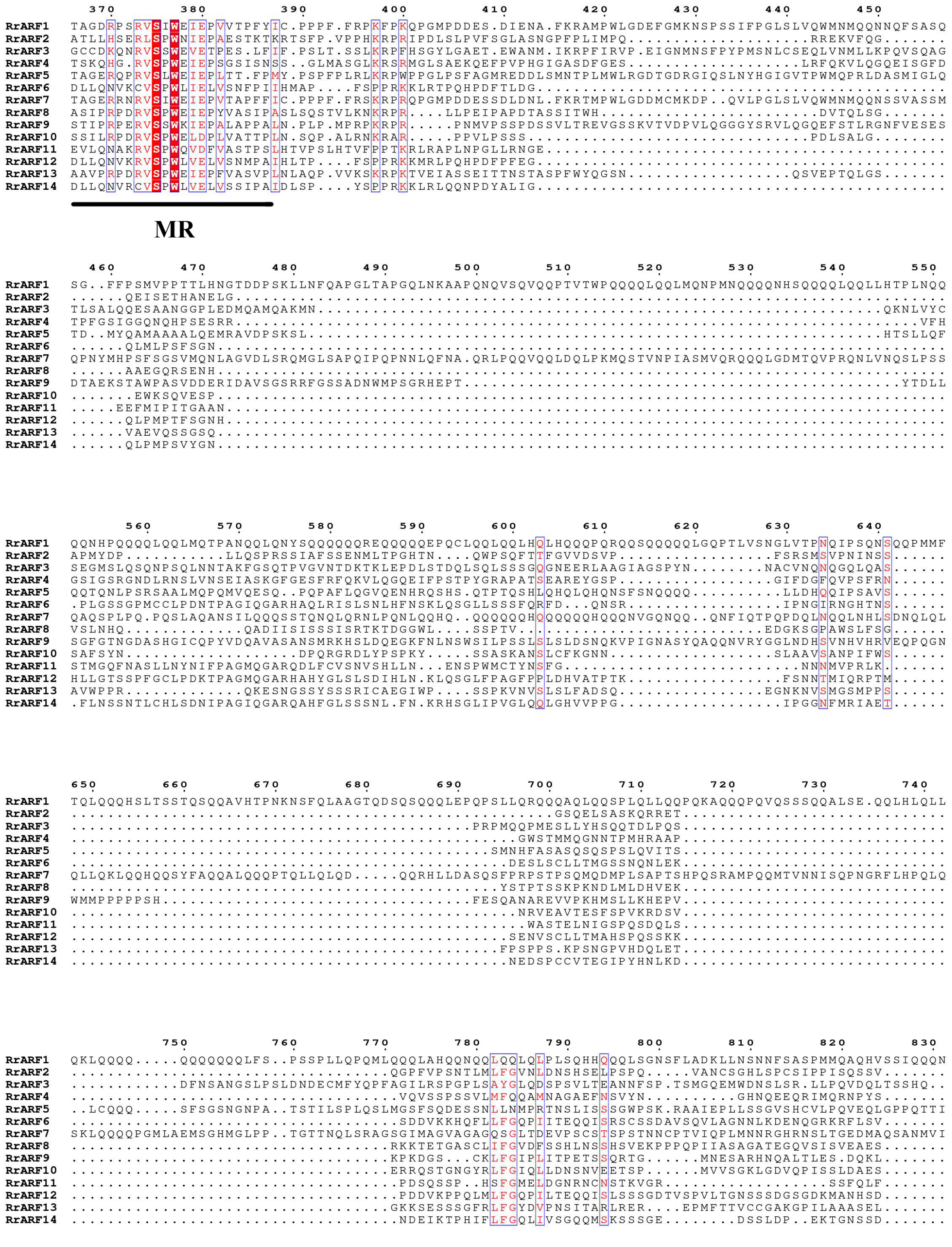
MR

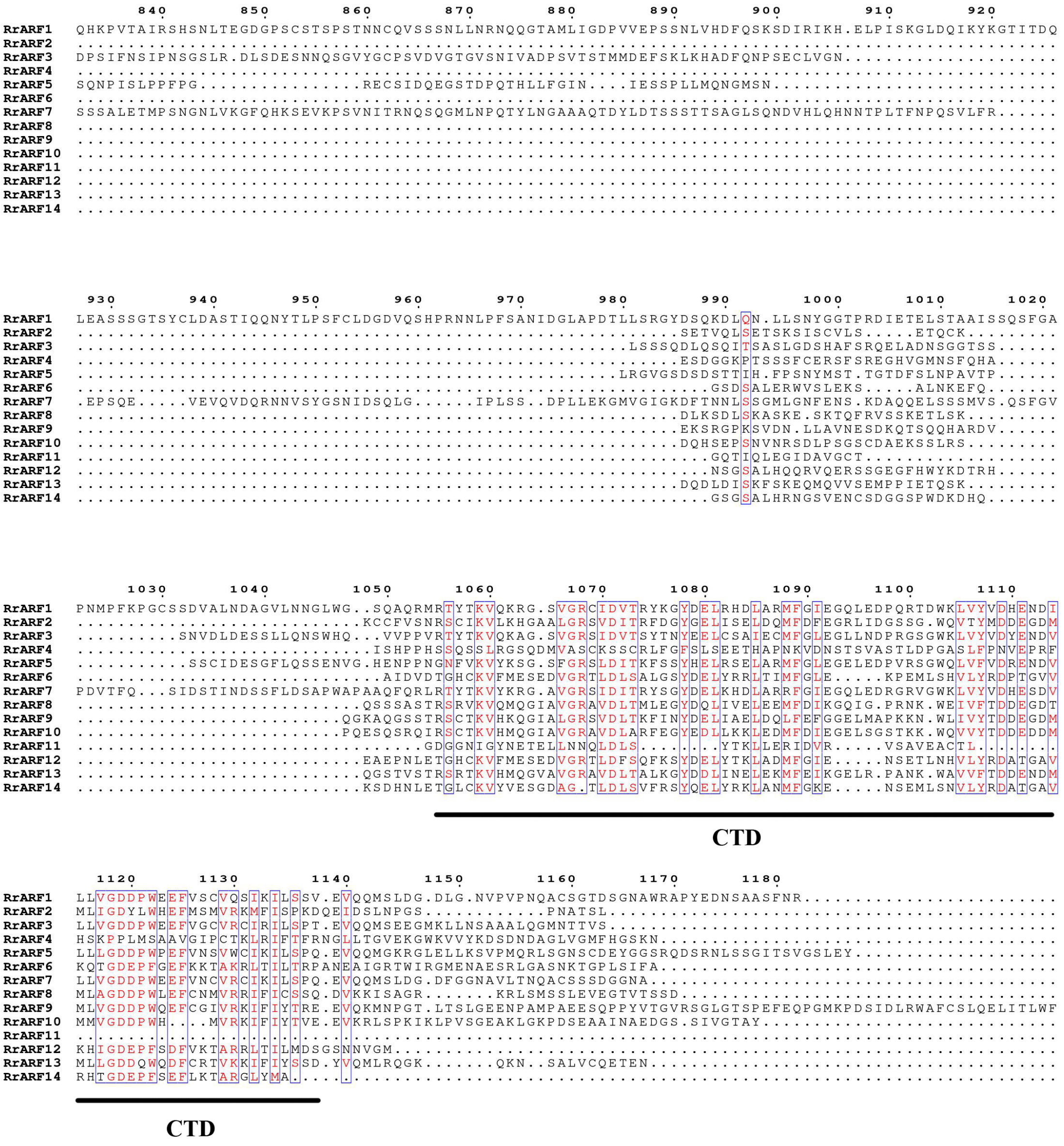

Figure S1. A comparison of the partial amino acid sequence of RrARFs. Black lines indicate conserved DBD structural domains、MR structural domains、CTD structural

domains .

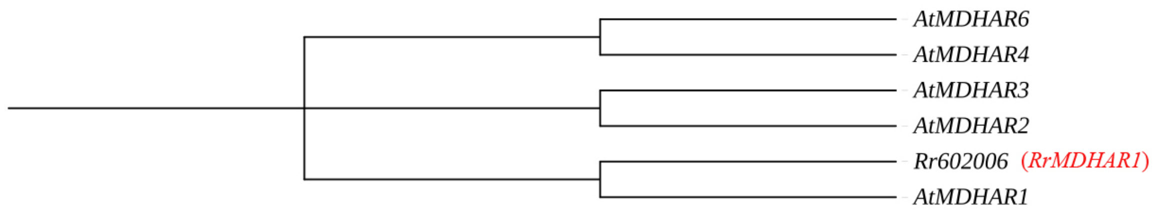

Figure S2. Phylogenetic tree of the MDHAR gene in *Arabidopsis thaliana*.

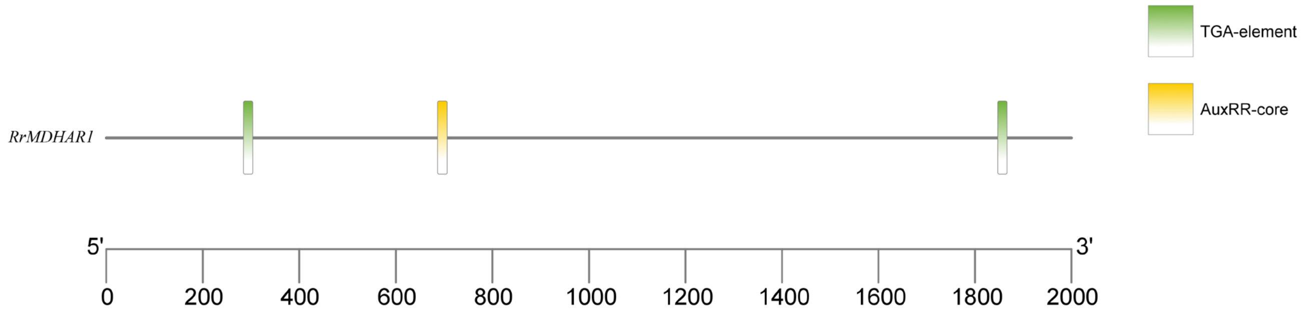

Figure S3. The cis-acting regulatory elements of *RrMDHAR1* gene in *R. roxburghii*.

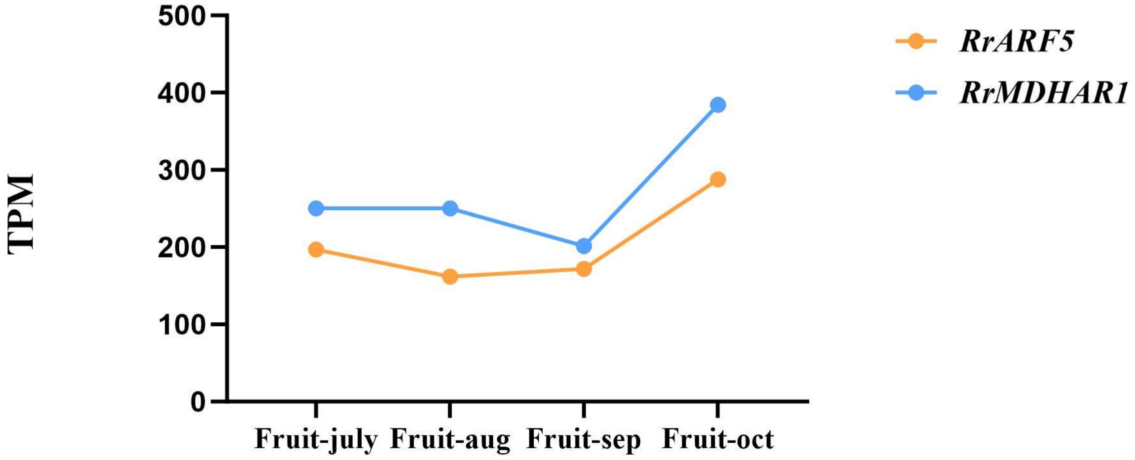

Figure S4. Transcription levels of *RrARF5* and *RrMDHAR1* at different fruit ripening stages by RNA-seq analysis

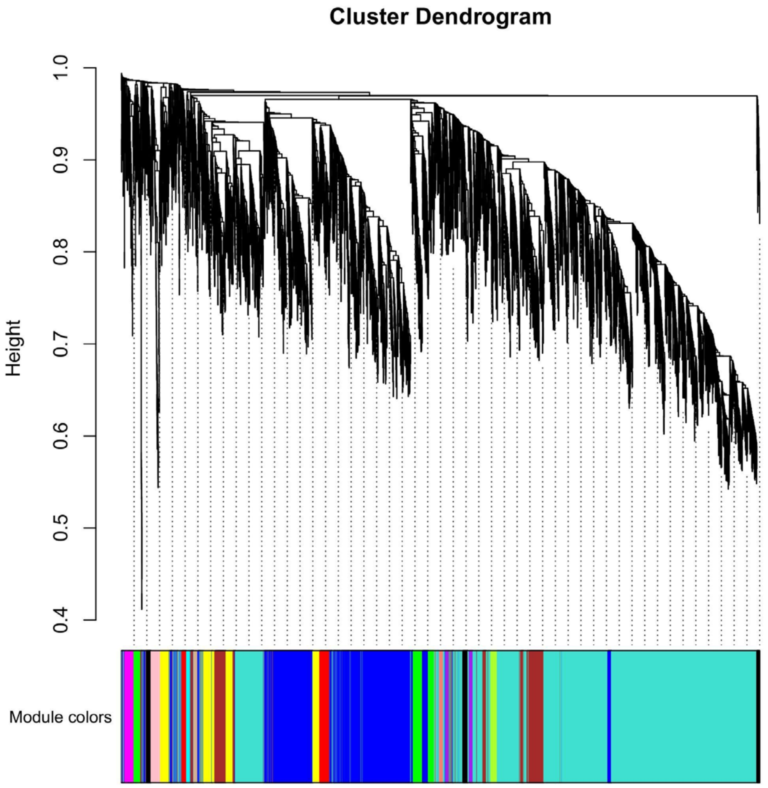

Figure S5. Hierarchical clustering tree with 14 co-expressed gene modules
